# A comparative study of machine learning models on molecular fingerprints for odor decoding

**DOI:** 10.1038/s42004-025-01651-7

**Published:** 2025-09-25

**Authors:** Jinyoung Suh, Yeonju Hong, Chunho Park

**Affiliations:** R&I Center, COSMAX Inc., Seongnam-si, Gyeonggi-do Republic of Korea

**Keywords:** Cheminformatics, Cheminformatics

## Abstract

Understanding how molecular structure relates to odor perception is a longstanding problem, with important implications for fragrance development and sensory science. In this study, we present an advanced comparative analysis of machine learning approaches for predicting fragrance odors, examining both individual descriptor‐based models and integrated frameworks. Using a curated dataset of 8681 compounds from ten expert sources, we benchmark functional group fingerprints, classical molecular descriptors, and Morgan structural fingerprints across Random Forest, eXtreme Gradient Boosting, and Light Gradient Boosting Machine. The Morgan-fingerprint-based XGBoost model achieves the highest discrimination (AUROC 0.828, AUPRC 0.237), outperforming descriptor-based models. Our findings highlight the superior representational capacity of molecular fingerprints to capture olfactory cues, not only achieving high predictive performance but also revealing a continuous, interpretable scent space that aligns with perceptual and chemical relationships. This paves the way for data-driven research into olfactory mechanisms, alongside the next generation of in silico odor prediction.

## Abstract (web summary)

Predicting fragrance odors remains a complex computational challenge. Here, the authors benchmark machine learning models using a curated dataset and underscore the potential of advanced fingerprints for data-driven fragrance design and enhanced in silico odor prediction.

## Introduction

The sense of smell, a fundamental aspect of communication and survival, transcends the boundaries of species, connecting humans, animals, and even plants through a shared chemical language. In humans, olfactory receptors not only reside in the nose but are distributed throughout the body, suggesting a multifaceted role in perception and interaction^1^. From early human history, fragrances have been used for religious and therapeutic purposes, and to mask unpleasant odors and enhance beauty. Despite this long-standing relationship, the exact mechanism of olfaction remains one of science’s enduring mysteries, intriguing researchers across disciplines. The first major revolution in olfactory research came with the discovery of olfactory receptor genes by Linda Buck and Richard Axel in 1991^2^, an achievement that earned them the Nobel Prize in Physiology or Medicine in 2004. This discovery provided the molecular framework of the olfactory system, revealing a combinatorial coding mechanism by which odorants interact with a diverse repertoire of receptors to create an astonishing array of smells. This foundational work sparked decades of exploration into the intricate relationship between molecular structures and sensory perception. In recent years, the integration of cheminformatics and machine learning (ML) has ushered in the second revolution in olfactory science. Researchers have developed computational models that bridge molecular structure and olfactory perception by leveraging diverse datasets, features, and predictive algorithms. Significant progress has been made in this field. In 2016, Nozaki and Nakamoto^3^ developed a predictive model based on an artificial neural network with a deep structure, using mass spectra of chemicals to predict odor impressions with notable accuracy (*R* ≈ 0.76). Their method addressed the inherent nonlinearity of the biological olfactory system, outperforming conventional linear models. In 2017, Shang et al.^4^ proposed a proof-of-concept machine-learning-based olfactometer to overcome the subjectivity and cost of human panelists in traditional gas chromatography/olfactometry. They built prediction models using molecular parameters with support vector machines (SVMs) and the Boruta algorithm for feature extraction, achieving high predictive accuracy (97.08%) for specific odor descriptors. In 2021, Sharma et al.^5^ advanced the field by leveraging deep neural networks (DNNs) and convolutional neural networks (CNNs) to decode the structure-odor relationship (SOR) from chemical compounds. They used physicochemical properties, molecular fingerprints, and 2D chemical images, with their Xception-based CNN model achieving predictive accuracies as high as 98.3%. In 2022, Saini and Ramanathan^6^ directly addressed the quantitative structure–odor relationship (QSOR) using multi-label classification strategies to predict odor from molecular structure. They curated a large dataset and explored techniques like binary relevance and classifier chains, finding that a binary relevance model with Daylight fingerprints yielded strong results. In 2023, Schicker et al.^7^ introduced Olfactory Weighted Sum (OWSum), a linear classification method relying solely on structural patterns (chemical fragments) as features. OWSum not only predicts odor but also provides insights into underlying structure-odor-relationships by assigning influence values to patterns, achieving a predicted accuracy of 67.7%. In 2024, Zhang et al.^8^ unveiled Molecular Representation by Positional Encoding of Coulomb Matrix (Mol-PECO), a deep learning model for QSOR that addresses limitations of conventional graph neural networks (GCNs). Mol-PECO leverages the Coulomb matrix and Laplacian eigenfunctions for positional encoding to capture molecular electrostatics and detailed structural information, outperforming traditional ML methods and GCNs. These advancements, summarized in Table 1, demonstrate significant progress in predictive olfactory models while also highlighting the persistent challenges of fully capturing the multidimensional complexity of odorant properties. In this study, we benchmarked various feature representations, including functional group (FG) fingerprints, classical molecular descriptors (MD), and Morgan structural fingerprints across diverse ML algorithms such as Random Forest (RF), XGBoost (XGB), and LightGBM (LGBM). Our findings revealed that the Morgan-fingerprint-based XGB model achieved the highest discrimination, demonstrated by an area under the receiver operating curve (AUROC) of 0.828 and area under the precision–recall curve (AUPRC) of 0.237, consistently outperforming descriptor-based models.

**Table 1:** Summary of recent machine learning approaches for odor prediction

Study	Machine learning model	Number of odorants	Feature source	Performance metrics	Reference number
Nozaki, Y. & Nakamoto, T. (2016)	Deep ANN	121	Mass spectral data	*R* ≈ 0.76	^3^
Shang, L. et al. (2017)	SVM (Boruta-C), ELM (PCA)	1026	DRAGON Physicochemical Parameters (PCA/Boruta)	SVM Accuracy: 97.08% ELM Accuracy: 97.53 ± 1.35%	^4^
Sharma, A. et al. (2021)	DNN (PPMF), CNN (Xception on 2D images)	5185	PaDel fingerprints; RDKit 2D chemical images	DNN Accuracy: 97.3% CNN Accuracy: 98.3% Combined Precision: 100% (64 smells)	^5^
Saini, K. & Ramanathan, V. (2022)	Daylight-BR	7374	Mordred, Morgan, Daylight fingerprints	micro-F1: 0.3523	^6^
Schicker, D. et al. (2023)	Olfactory Weighted Sum (linear)	64	SMARTS structural patterns	Predicted Accuracy: 0.677 Training Accuracy: 0.905 Random Guessing Performance: 0.214	^7^
Zhang, M. et al. (2024)	Mol-PECO (Deep Learning, Coulomb Matrix/LPE)	8503	Coulomb matrix + LPE encodings	AUROC: 0.813 AUPRC: 0.181	^8^
This study	RF, XGB, LGBM	8681	Morgan fingerprints (ST Model)	XGB-ST AUROC: 0.828 XGB-ST AUPRC: 0.237 LGBM-ST AUROC 0.810 LGBM-ST AUPRC 0.228	Table 2

Overview of major studies applying machine learning (ML) models to olfactory prediction tasks. Shown are the model types, number of odorants used for training, feature sources, and key performance metrics.

*ANN* artificial neural network, *R* Pearson correlation coefficient, *SVM* support vector machine, *ELM* extreme learning machine, *PCA* principal component analysis, *CNN* convolutional neural network, *DNN* deep neural network, *PPMF* physiochemical properties and molecular fingerprints, *BR* binary relevance, *SMARTS* SMILES arbitrary target specification, *LPE* learned positional encoding, *ST* structural (Morgan) fingerprint, *AUROC* area under the receiver operating characteristic curve, *AUPRC* area under the precision–recall curve, *RF* Random Forest, *XGB* eXtreme Gradient Boosting, *LGBM* Light Gradient Boosting Machine.

This research underscores the superior capacity of topological and conformational fingerprints to effectively capture olfactory cues, thereby paving the way for data-driven fragrance design and the next generation of in silico odor prediction.

## Methods

### Dataset

We assembled a comprehensive human olfactory perception dataset by unifying ten expert-curated sources, all accessed via the pyrfume-data GitHub archive (https://github.com/pyrfume/pyrfume-data)^9^. The individual contributions were: Arctander’s dataset, AromaDb, FlavorDb (odor), FlavorNet, The Good Scents Company Information System (TGSC), the International Fragrance Association (IFRA) Fragrance Ingredient Glossary, Leffingwell’s compendium, Sharma_A, Sharma_B, and Sigma’s Fragrance & Flavor Catalog. Starting from this raw archive, we carried out a rigorous multistep refinement to produce an analysis-ready matrix of 8681 unique odorants and 200 candidate descriptors (100 with ≥ 30 occurrences). Starting from the ten source datasets, we first merged them into a single unified table keyed by PubChem CID. For each CID, we listed all raw descriptor labels in full without deduplication and retrieved the canonical Simplified Molecular Input Line Entry System (SMILES) via PubChem’s PUG-REST API^10^. We then prioritized the three odor descriptors provided by the IFRA (Fragrance Ingredient Glossary, April 2020) as the most trusted descriptors^11^, supplementing them with commonly used terms (e.g., Fishy, Odorless) to define a 201-label set (200 labels plus “Others”). Because the original ten datasets contained inconsistencies—such as leading/trailing whitespace, typographical errors, language variants, and subjective terms—we standardized every descriptor to one of these controlled 201 labels under the guidance of perfumery experts, thereby yielding a fully curated multi-label dataset ready for ML.

### Feature extraction

FG features for the **FG model** were generated by detecting predefined substructures using SMARTS patterns^12^. A full list of FGs and their corresponding SMARTS definitions is provided in Supplementary Table 1. Molecular features for the **MD model** were calculated using the RDKit library^13^. These features included molecular weight (MolWt), number of hydrogen donors and acceptors, topological polar surface area (TPSA), molecular logP (molLogP), number of rotatable bonds, heavy atom count, and ring count. For the **ST model**, molecular fingerprints were derived using the Morgan algorithm^14^ from MolBlock representations^15^. MolBlock representations were generated from SMILES^16^ strings and optimized using the universal force field algorithm^17^ to ensure chemically valid conformations.

### Modeling odor complexity

Unlike simple binary classification, all models support multi-label classification, reflecting the complex and overlapping nature of olfactory descriptors. For instance, a molecule can simultaneously exhibit “Floral” and “Spicy” characteristics. ML classifiers are trained for each odor class, leveraging the multi-dimensional fingerprints to capture non-linear relationships between structural features and odor labels. Labels are binarized using a MultiLabelBinarizer, which encodes the presence or absence of each odor category.

### Model development and evaluation

We benchmarked three tree-based algorithms: (i) RF^18^, selected for its interpretability and robustness to class imbalance; (ii) XGB^19^, leveraging second-order gradient optimization and L1/L2 regularization to excel on sparse, high-dimensional fingerprints; and (iii) LGBM^20^, employing leaf-wise tree growth and histogram-based splitting for fast, memory-efficient training on large descriptor sets. For each algorithm and each descriptor combination, separate one-vs-all classifiers were trained per odor label. To ensure reliable generalization estimates, we performed stratified fivefold cross-validation on an 80:20 train:test split, maintaining the positive:negative ratio within each fold. Within each fold, models were fitted on four subsets and evaluated on the held-out subset, yielding mean metrics across folds: Accuracy (fraction correctly classified), AUROC, AUPRC, Specificity (true negative rate), Precision(proportion of positive predictions that are correct), and Recall (proportion of actual positives that are correctly identified).

## Results

In the comparative evaluation of model performance, we assessed nine combinations of three feature sets—FG, MD, and ST—with three tree-based classifiers (RF, XGB, and LGBM). A complete breakdown of performance evaluation across all odor labels is available in Supplementary Data 1. Across all tested configurations, XGB consistently demonstrated the strongest results regardless of feature set. Notably, the ST-XGB model achieved the most favorable trade-off between discrimination and retrieval, with an AUROC of 0.828 and an AUPRC of 0.237, as well as 97.8% accuracy, 99.5% specificity, 41.9% precision, and 16.3% recall. For comparison, MD-XGB achieved an AUROC of 0.802 and an AUPRC of 0.200, while FG-XGB attained lower values (AUROC = 0.753, AUPRC = 0.088). Although RF and LGBM paired with Morgan fingerprints also performed robustly (ST-RF: AUROC = 0.784, AUPRC = 0.216; ST-LGBM: AUROC = 0.810, AUPRC = 0.228), neither surpassed the combined ranking of ST-XGB. These results underscore that structure-derived fingerprints are highly effective in capturing olfactory cues, and that gradient-boosted decision trees—particularly XGB—are well suited to leveraging this information for accurate multi-label odor prediction (Table 2).

**Table 2:** Test‐set performance of feature sets and classifiers

Feature set	Classifier	Accuracy	AUROC	AUPRC	Specificity	Precision	Recall
FG	LGBM	0.696	0.747	0.088	0.695	0.056	0.693
	RF	0.733	0.736	0.086	0.732	0.061	0.648
	XGB	0.977	0.753	0.088	0.998	0.071	0.018
MD	LGBM	0.947	0.800	0.197	0.955	0.169	0.353
	RF	0.967	0.730	0.172	0.981	0.221	0.234
	XGB	0.977	0.802	0.200	0.995	0.329	0.131
ST	LGBM	0.959	0.810	0.228	0.968	0.215	0.327
	RF	0.974	0.784	0.216	0.989	0.279	0.187
	XGB	0.978	0.828	0.237	0.995	0.419	0.163

Classification performance of Random Forest (RF), eXtreme Gradient Boosting (XGB), and Light Gradient Boosting Machine (LGBM) models using three types of molecular representations: functional group descriptors (FG), molecular descriptors (MD), and structural Morgan fingerprints (ST). Reported metrics include accuracy, area under the receiver operating characteristic curve (AUROC), area under the precision–recall curve (AUPRC), specificity, precision, and recall. All metrics are averaged across binary classification tasks for odor labels represented by 30 or more samples.

Fivefold cross-validation further confirmed the robustness of our findings, with the ST-XGB model again exhibiting superior performance among the nine candidate combinations. Detailed cross-validation results are available in Supplementary Table 2. In this setting, ST-XGB achieved a mean AUROC of 0.816 and AUPRC of 0.226, outperforming both ST-RF (AUROC 0.784, AUPRC 0.215) and ST-LGBM (AUROC 0.801, AUPRC 0.224), while maintaining high specificity (>99%) and precision (>35%) without a substantial drop in recall (~14%). These cross-validation results underscore the consistency and generalizability of the fingerprint-based XGB model for olfactory prediction. In the field of odor prediction ML, reported accuracies frequently exceed 90%, yet such figures can be misleading when most odor categories are severely imbalanced. Although our ST-XGB model attains an overall accuracy of 0.978, accuracy alone fails to capture performance on rare odor labels. Instead, we focus on threshold-independent metrics—AUROC and AUPRC—which better reflect true predictive power across all classes. Remarkably, these results derive solely from straightforward Morgan fingerprints, without resorting to more elaborate encodings, and they match or exceed those of complex alternatives. Fivefold cross-validation further confirms the robustness of this simple fingerprint plus gradient-boosting strategy.

### Modeling and performance summary for the 9 selected odor labels

We focus on nine carefully selected odor labels—CITRUS, FRUITY, GREEN, FLORAL, WOODY, MUSK, EARTHY, ODORLESS, and MEATY—which together span the key notes most prized by perfumers and formulators. Among these, CITRUS, FRUITY, GREEN, FLORAL, WOODY, and MUSK notes were chosen as they represent the fundamental classifications in the fragrance industry, forming the backbone of perfumery for their widespread use in fine fragrances and personal-care products. The inclusion of EARTHY reflects the recent global surge in unisex and niche fragrances, where mossy, soil-like accords lend depth and universality to contemporary scent blends. Meanwhile, growing “chemophobia” in the cosmetics industry has spurred demand for truly neutral or odor‐free ingredients: consumers now expect their lotions and creams to carry a clean, unadulterated feel, yet raw‐material suppliers often describe odor only in vague “characteristic” terms. By incorporating ODORLESS as a target label, we enable fragrance researchers and product developers to rigorously screen for—and certify—the absence of unwanted notes. Finally, heightened interest in plant-based and laboratory-grown “meat alternatives” has placed unprecedented emphasis on olfactory authenticity in alternative-protein foods. Before even tasting, consumers judge such products by their aroma; a convincing “meaty” note is therefore vital to adoption. Including MEATY among our target labels allows us to address this emerging frontier, equipping both flavor scientists and perfumers with the tools to predict—and ultimately design—the olfactory profiles that will define next-generation consumables.

**Fig. 1:**
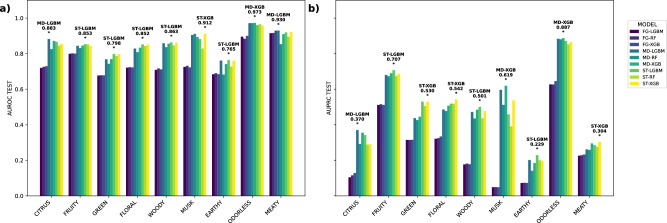
Performance comparison of machine-learning models across odor labels. **a** shows the area under the receiver operating characteristic curve (AUROC), and **b** shows the area under the precision–recall curve (AUPRC) for each label across nine selected models. Asterisks indicate the best-performing model per label. Colors correspond to model types (see legend in figure). The numerical data underlying this figure are available in Supplementary Data 1.

While ST-XGB emerged as the top‐performing model on average across all odor labels, a label‐by‐label analysis reveals a more nuanced landscape of optimal classifiers. Notably, the FG feature group—relying solely on the presence or absence of FGs—consistently yielded the lowest predictive performance, underscoring that simple SMARTS‐based fingerprints alone cannot fully capture the complexity of olfactory profiles. Nonetheless, FG‐based models did perform surprisingly well for the MEATY note, where FG-XGB attained the highest AUROC (0.929) among three FG classifiers for 9 labels. By contrast, MD frequently led the field for individual notes. MD-LGBM achieved the best results for CITRUS (AUROC = 0.883; AUPRC = 0.370), while MD-XGB dominated ODORLESS (AUROC = 0.973; AUPRC = 0.887). MD-LGBM also recorded an AUROC of 0.930 on MEATY, and MD-XGB delivered an AUPRC of 0.619 for MUSK—further evidence that physicochemical descriptors provide powerful, complementary information. Although the ST feature group generally outperformed FG and MD, the leading tree‐based algorithm varied by note: within ST, LGBM often surpassed both XGB and RF, demonstrating that even for structurally rich representations, algorithmic choice can yield incremental gains (Fig. 1).

### Functional group (FG) models

**Fig. 2:**
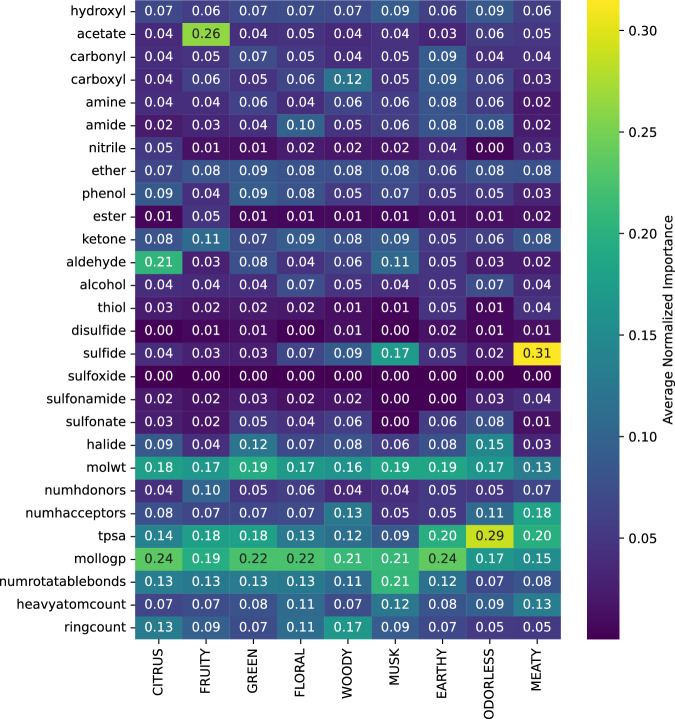
Feature importances across odor labels. Heatmap showing the average normalized feature importances of functional‐group and molecular descriptors across Random Forest (RF), eXtreme Gradient Boosting (XGBoost), and Light Gradient Boosting Machine (LightGBM) models for each odor label. The numerical data underlying this figure are available in Supplementary Data 2.

To robustly assess the contribution of each FG, we computed consensus feature importances by averaging across the three tree-based MLs (Fig. 2). Three functional‐group motifs stand out as primary drivers of olfactory classification. First, the acetate pattern dominates the FRUITY axis with an average importance of 0.26, a finding that resonates with the known roles of isoamyl acetate and ethyl butyrate in banana- and pineapple-type aromas. Second, aldehyde functionality registers a peak importance of 0.21 in the CITRUS profile, reflecting the sharp, zesty accents imparted by octanal and citral in lemon and orange accords. Finally, sulfide moieties achieve an importance of 0.31 for the MEATY note, consistent with the central role of thiol- and sulfide-containing volatiles in cooked-meat and savory character. These three FG signals capture the most salient structure–odor relationships, while other SMARTS patterns contribute minimally to sensory discrimination.

### Molecular descriptor (MD) models

MD importance analysis unambiguously identifies three physicochemical descriptors—molLogP, MolWt, and TPSA—as principal determinants of odor classification (Fig. 2). molLogP (mean importance ≈ 0.206) underscores the pivotal role of lipophilicity in driving high‐affinity interactions within the hydrophobic binding crevice of olfactory G protein–coupled receptors (GPCRs). MolWt (mean importance ≈ 0.172) emerges not only as a key predictor of volatility, delineating the distinction between bright “top notes” and more persistent “base notes,” but also serves as a practical proxy for molecular size. Because MolWt is directly proportional to molecular size, it provides a useful gauge for whether a compound can physically fit into the binding site of an olfactory receptor. TPSA (mean importance ≈ 0.170) reflects the critical balance between mucosal diffusion and receptor-site polarity, with excessively polar compounds naturally failing to elicit a perceptible scent. Far from being mere modeling artifacts, these findings deliver actionable guidance for fragrance design, and we are poised to validate their mechanistic underpinnings in targeted follow-up studies.

**Fig. 3:**
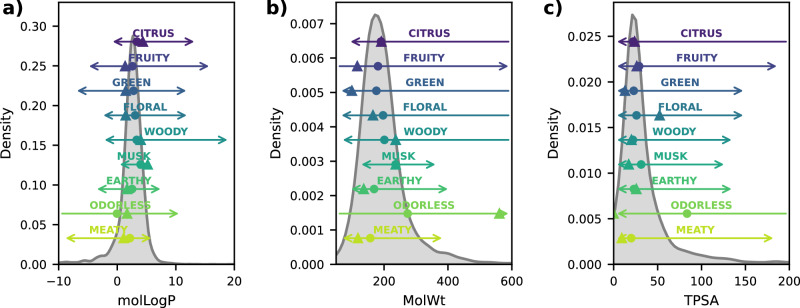
Comparative raw and model‐optimal distributions of three molecular descriptors (MD). Kernel density estimates for **a** molecular Log P, **b** molecular weight, and **c** topological polar surface area (TPSA). Each plot overlays class-specific min–max ranges and mean values (●), alongside model-optimized values (▲) derived from the molecular descriptor-based eXtreme Gradient Boosting (MD-XGB) model for each odor label. Mean points (●) represent average descriptor values of all raw samples with that label; model-optimal points (▲) represent the most confident predictions per odor label. The numerical data underlying this figure are available in Supplementary Data 3.

Building on the descriptor‐importance results, we next turn our attention to the underlying physicochemical property distributions—and how these raw profiles compare to the model‐optimized exemplars for each fragrance class (Fig. 3a–c, “Raw vs. Model-Optimal”). First, we compute and visualize the raw molLogP, MolWt, and TPSA distributions across all compounds in our dataset, overlaying kernel density estimates to capture the overall chemical space occupied by each odor labels. We then overlay minimum–maximum ranges (with the mean indicated by solid points) for each odor label to highlight the natural variability inherent in the data. Finally, by training an MD-XGB classifier on the same three descriptors and identifying, for each class, the test sample with the highest predicted probability, we pinpoint the “model-optimal” compound that best represents the most discriminative physicochemical signature. Comparing these optimal points against the raw distributions not only validates the importance rankings from Fig. 2 but also refines our understanding of each fragrance class by illustrating where the model focuses its predictive power within the broader chemical landscape.

**Molecular LogP (**Fig. 3a). Both the raw mean and the model-optimal value for musk occupy the highest LogP region of the dataset (≈ 5.2), confirming that musk compounds are the most lipophilic. This elevated lipophilicity underlies their pronounced skin persistence and affinity for the hydrophobic crevice of olfactory GPCRs and explains why musks serve as enduring base notes in perfumery. Interestingly, citrus notes also lie in a relatively high LogP domain (raw mean ≈4.4), owing to the abundance of non-polar monoterpenes that feature unsaturated hydrocarbon frameworks. In ethanol-based fine fragrance formulations, this lipophilicity can even contribute to turbidity over time. At the opposite extreme, the odorless class exhibits the lowest mean LogP (≈ 1.1) with outliers descending below −10. Such low lipophilicity prevents traversal of the nasal mucus lipid layer, precluding receptor engagement and resulting in an absence of perceived scent. **Molecular Weight (**Fig. 3b). Woody and musk categories (excluding odorless) occupy the highest molecular‐weight regime (≈230–240 Da), consistent with their roles as persistent base notes. Unexpectedly, citrus notes appear at the upper end of the top‐note spectrum (≈190 Da), illustrating that many green and fruity volatiles dissipate almost immediately upon blotter application (e.g., grass alcohols), whereas some citrus compounds persist significantly longer. The odorless class again defies typical olfactory wisdom by spanning to the highest weights (>300 Da), suggesting that effective scent molecules must remain below this threshold to access the binding pocket of olfactory receptors. **Topological Polar Surface Area** (Fig. 3c). Odorless compounds possess the highest mean TPSA (≈ 55 Å²), reflecting extreme polarity that inhibits passive diffusion through the lipid-rich nasal mucosa and perhaps fails to satisfy the predominantly hydrophobic binding site of GPCR odorant receptors. Paradoxically, the model-optimal TPSA for odorless falls to near zero, indicating that the classifier has also learned that minimally polar (TPSA ≈ 0) molecules—unable to solubilize in the aqueous mucus layer—likewise never reach receptor sites and thus register as odorless.

### Structural (ST) models

Recent approaches^8^ have utilized high-dimensional Coulomb matrix descriptors to capture pairwise electrostatic interactions, but our streamlined pipeline employs only 2D Morgan fingerprints derived from 3D MolBlock geometries. This strategy markedly reduces computational demands while still effectively capturing key substructural motifs. Classification performance with these fingerprints was found to match, and in some cases exceed that of Coulomb matrix–based models. Although the XGB-based model (ST-XGB) achieved the highest mean AUROC, its lower recall led us to emphasize the LightGBM variant (ST-LGBM) for subsequent label-specific analyses, where maximizing recall is critical for comprehensive odor profiling and safety. For each of the nine odor labels, we identified the single test-set compound to which ST-LGBM assigned the highest probability. This analysis highlights the molecular scaffolds to which the model is most sensitive—such as aliphatic esters for fruity, terpenes for citrus, and fused tricyclic frameworks for woody notes. These representative motifs align closely with established fragrance chemotypes, demonstrating that simple 2D fingerprints can reliably capture essential structural features underlying olfactory perception. The strong correspondence between these high-confidence selections and known fragrance scaffolds, as summarized in Table 3, underscores the robustness of 2D molecular fingerprints as a foundation for high-throughput odor prediction and rational fragrance design. The superior performance of the ST model can be attributed to the structural expressiveness of Morgan fingerprints, which represent atom-level topological environments with high resolution. Although these fingerprints are inherently two-dimensional and encode only connectivity, we derive them from molecules that have been pre-optimized in three dimensions using force-field methods. This preprocessing ensures chemically valid input structures by standardizing hydrogen placement, bond configuration, and tautomeric forms. As a result, the model benefits from both the representational power of topological substructures and the consistency of optimized molecular inputs, allowing tree-based classifiers to more effectively learn odor-relevant motifs and distinguish subtle structural differences among fragrance compounds. To further improve prediction robustness and capture diverse perspectives within the ST model family, we developed an ensemble prediction framework that integrates three tree-based classifiers trained on Morgan fingerprints: ST-RF, ST-XGB, and ST-LGBM.

**Table 3:** Representative test-set compounds with highest model-predicted probabilities for each odor label

Odor label	Compound CID	Compound name	Assigned odor descriptors	Probability
CITRUS	20797	Nookatone	CITRUS, FRUITY, GRAPEFRUIT	0.974
FRUITY	16324	Allyl butyrate	FRUITY, APRICOT, PINEAPPLE	0.973
GREEN	324382	Methyl 2-decynoate	WAXY, NUTTY, GREEN	0.977
FLORAL	10176245	(5R)-2,5,6-trimethylheptan-2-ol	FLORAL, TERPENIC, ROSE	0.972
WOODY	24758199	Methyl cedryl ether	GREEN, WOODY, CINNAMON	0.984
MUSK	71332160	4-tert-butyl-2,6-dimethyl-3,5-dinitrobenzaldehyde	MUSK	0.997
EARTHY	32065	Nutty pyrazine	EARTHY, OTHERS, ROASTED	0.973
ODORLESS	135565913	Dipotassium guanylate	ODORLESS	0.999
MEATY	47649	2-methyl-3-(methyldisulfanyl)furan	OTHERS, CHEMICAL, MEATY	0.995

For each odor label, the ST-LGBM model selected the test-set compound with the highest predicted probability. In all cases, the predicted label was among the compound’s assigned odor descriptors, suggesting consistency between model output and expert annotation.

*CID* PubChem compound identifier, *ST* structural (Morgan) fingerprint, *LGBM* Light Gradient Boosting Machine.

### Ensemble framework for olfactory prediction

For every candidate molecule, we compute its probability for each odor label in each model, then simply pick the three highest‐scoring label–model pairs. By focusing on these three “ST” models rather than all nine possible combinations, we retain interpretability and improve precision. As a quick sanity check, we re‐evaluated three well–characterized fragrance standards from our training set. Linalool (CAS 78-70-6) came out as Lavender (0.999 in ST-LGBM), Petitgrain (0.998, ST-LGBM), and Floral (0.980, ST-RF)—exactly matching its known dual role in lavender oil and other floral terpenoids. Limonene (5989-27-5) was predicted correctly as Terpenic (0.988, ST-LGBM), Pine (0.963, ST-LGBM), and Citrus (0.918, ST-LGBM), capturing both its citrus zest and conifer‐like notes. Phenyl Ethyl Alcohol (60-12-8) emerged as Honey (0.986, ST-LGBM), Rose (0.966, ST-LGBM), and Floral (0.940, ST-RF), reflecting its classic rose scent. We then inputted three cosmetic raw materials. Isoamyl Laurate is added to skin care products for its silky skin feel, but can sometimes betray an alcoholic and greasy odor. Originally labeled in our data only as Fatty, Oily, and Alcoholic, our ensemble reinterpreted it as Brandy (0.983, ST-LGBM), Apricot (0.971, ST-LGBM), and Alcoholic (0.950, ST-LGBM), neatly describing its characteristic odor. Glutathione (70-18-8) inherently contains an SH group—and can smell like rotten egg—but our model interestingly predicted it as Yeast (0.999, ST-LGBM), suggesting a fermentation‐like nuance rather than raw sulfur. Squalane (111‑01‑3), which is virtually odorless and was not present in our dataset, was nevertheless predicted as Fishy (0.983, ST‑LGBM). Interestingly, its unsaturated analogue, squalene, was included in the dataset and labeled as Floral and Oily, with no association to Fishy descriptors. However, squalene is known to oxidize into aldehydes with fishy odor characteristics. The prediction of “Fishy” for odorless squalane suggests that the ST‑LGBM model may infer latent odor-relevant features based on structural similarity and external chemical behavior. Finally, we screened all 400 dipeptides constructed from the twenty amino acids and highlighted the two strongest Meaty candidates. Gln-Met scored Meaty (0.892, ST-LGBM), Savory (0.689, ST-XGB), and Cheesy (0.647, ST-XGB) without invoking any Sulfurous note—making it a prime flavor precursor. This aligns with Damian et al.’s finding^21^ that free Met contributes directly to meatiness. Pro-Ala achieved Meaty (0.747, ST-LGBM). Because it lacks Met’s sulfur, it avoids overt off‐smells yet still delivers a high meaty probability, suggesting it could be a “clean” meaty peptide. We also noted a dipeptide, Pro-Phe with a strong Floral score—borderline odorless—that might even be explored as a novel fragrance ingredient. A summary of these predictions for fragrance standards, cosmetic materials, and dipeptides is provided in Table 4. In sum, this streamlined ST-only ensemble not only reproduces known scent descriptors for classic fragrance molecules but also generalizes well to cosmetic materials and uncovers new fragrance and flavor candidates from peptides. By concentrating on fingerprint‐driven models, we achieve equally interpretable, more precise predictions—provides a practical foundation for discovering novel fragrance and flavor ingredients across perfumery, cosmetics, and food industries. While the ensemble results demonstrate strong predictive performance, we further investigated whether the ST models learn deeper structural regularities among odor descriptors that go beyond discrete label assignments.

**Table 4:** Predicted odor profiles of fragrance standards, cosmetic raw materials, and dipeptides using structure-based classifiers

Category	CAS number	Compound name	Predicted label 1	Predicted label 2	Predicted label 3
Fragrance	78-70-6	Linalool	LAVENDER (0.999, ST-LGBM)	PETITGRAIN (0.998, ST-LGBM)	FLORAL (0.980, ST-RF)
	5989-27-5	Limonene	TERPENIC (0.988, ST-LGBM)	PINE (0.963, ST-LGBM)	CITRUS (0.918, ST-LGBM)
	60-12-8	Phenyl ethyl alcohol	HONEY (0.986, ST-LGBM)	ROSE (0.966, ST-LGBM)	FLORAL (0.940, ST-RF)
Cosmetic raw material	6309-51-9	Isoamyl laurate (skin-conditioning agents)	BRANDY (0.983, ST-LGBM)	APRICOT (0.971, ST-LGBM)	ALCOHOLIC (0.950, ST-LGBM)
	70-18-8	Glutathione (skin brightening)	YEAST (0.999, ST-LGBM)	ODORLESS (0.859, ST-LGBM)	MILD (0.419, ST-LGBM)
	111-01-3	Squalane (skin-conditioning agents)	FISHY (0.983, ST-LGBM)	WAXY (0.859, ST-LGBM)	CITRUS (0.854, ST-LGBM)
Dipeptide	114659-59-5	Gln-Met	MEATY (0.892, ST-LGBM)	SAVORY (0.689, ST-XGB)	CHEESY (0.647, ST-XGB)
	6422-36-2	Pro-Ala	MEATY (0.747, ST-LGBM)	ODORLESS (0.360, ST-RF)	OTHERS (0.325, ST-LGBM)
	13589-02-1	Pro-Phe	FLORAL (0.852, ST-LGBM)	ODORLESS (0.463, ST-LGBM)	FRUITY (0.200, ST-RF)

For each compound, the top three odor labels were predicted by ST-RF, ST-XGB, and ST-LGBM models trained on Morgan fingerprints. Odor descriptors for known substances reflect commonly recognized or empirically grounded characteristics. Dipeptides, not tested experimentally, were included as exploratory cases to illustrate potential in novel fragrance discovery.

*ST* structural (Morgan) fingerprint, *RF* Random Forest, *XGB* eXtreme Gradient Boosting, *LGBM* Light Gradient Boosting Machine.

### Mapping the learned odor topology

**Fig. 4:**
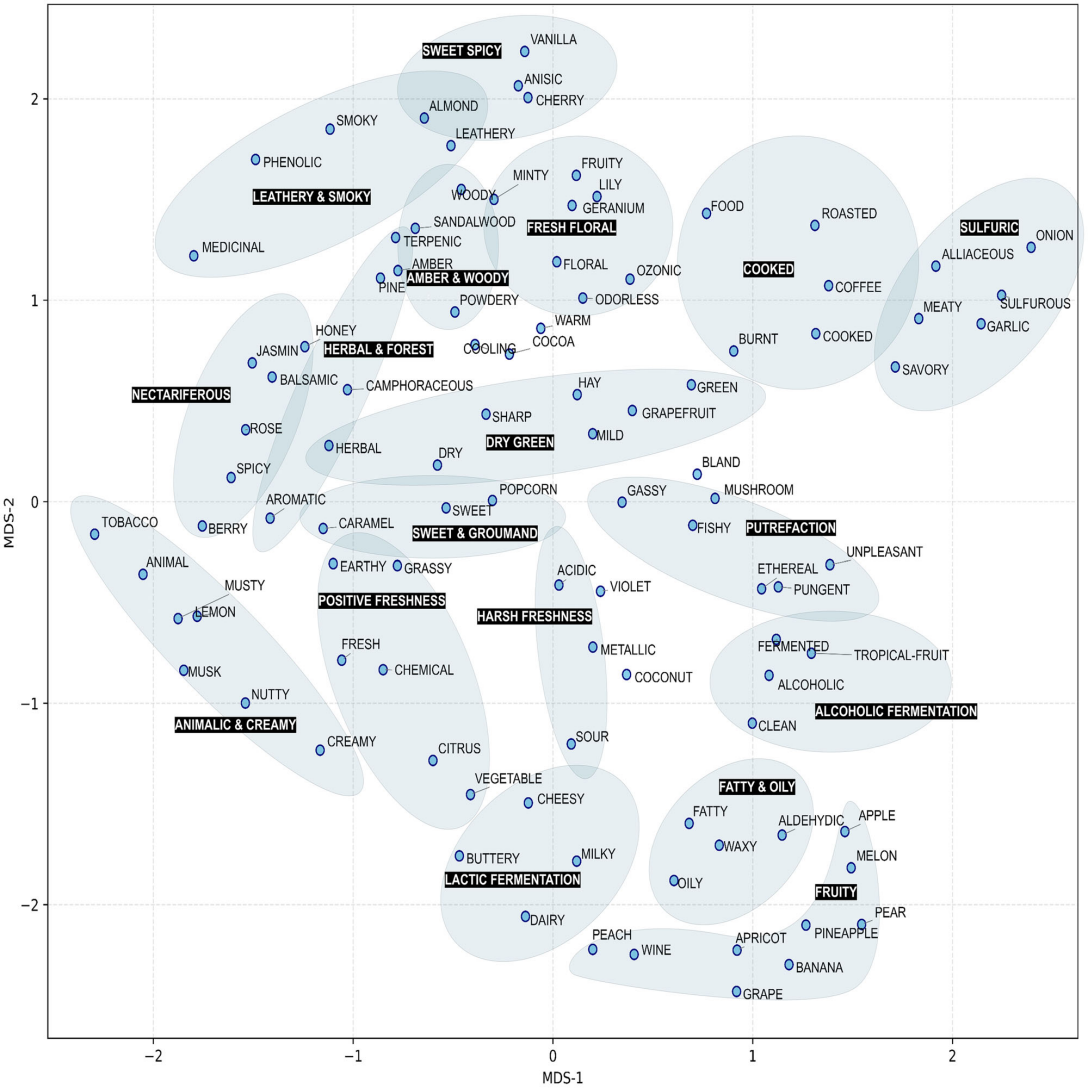
Two-dimensional MDS projection of inter-label distances derived from UMAP embeddings of fingerprint importance vectors. Odor label-specific feature importance vectors obtained from ST-LGBM classifiers were first embedded into a three-dimensional space using Uniform Manifold Approximation and Projection (UMAP) with a cosine distance metric. Euclidean distances between the embedded points were then calculated, and classical multidimensional scaling (MDS) was applied to generate a two-dimensional projection that preserves inter-label spatial relationships. Each point represents a single odor descriptor. This geometry-preserving visualization reveals functional and perceptual proximity among odor labels within the learned fingerprint space. The numerical data underlying this figure are available in Supplementary Data 4.

To explore latent relationships among odor labels, we computed pairwise distances between label-specific feature importance vectors obtained from independently trained ST-LGBM classifiers. These 1024-dimensional vectors were first embedded into a three-dimensional space using Uniform Manifold Approximation and Projection (UMAP)^22^ with a cosine distance metric. Euclidean distances among the embedded points were then calculated, followed by classical multidimensional scaling (MDS) to generate a two-dimensional projection that preserves global inter-label relationships (Fig. 4). Based on this projection, fragrance experts identified several distinct and interpretable clusters. For example, the “Lactic Fermentation” cluster includes odor labels such as buttery, cheesy, and dairy, which are strongly associated with milk-derived fermentation and fatty acids. In contrast, the “Alcoholic Fermentation” cluster contains clean, alcoholic, and fermented odor labels. While not spatially adjacent to Lactic Fermentation, it appears to lie along a perceptual gradient originating near Fruity, progressing toward ethanol-related impressions. Following this axis further, one encounters a more aversive region—the “Putrefaction” cluster—encompassing unpleasant, pungent, fishy, and gassy odor labels. In the lower part of the map, the “Sulfuric” cluster emerges, defined by odor labels such as onion, garlic, meaty, and alliaceous, which are commonly linked to sulfur-containing food volatiles. Adjacent to it is the “Cooked” cluster, characterized by roasted, burnt, and cooked odor labels, likely reflecting thermal transformations of sulfuric or protein-based compounds. At the upper region of the projection, a distinct axis of woody and forest-related odor labels becomes evident. The “Leathery & Smoky” cluster includes leathery and smoky, evoking charred wood impressions, while the neighboring “Amber & Woody” cluster includes woody and sandalwood, consistent with heartwood olfactory profiles. Adjacent to these are the “Herbal & Forest” clusters, which comprise terpenic, pine, and camphor odor labels, often associated with forest volatiles and phytoncides. Although partially informed by expert interpretation, these spatial arrangements exhibit perceptual continuity and chemical coherence aligned with known olfactory taxonomies. Notably, they reflect sensory transitions shaped by microbial degradation, thermal processes, and the chemical diversity of natural raw materials. Collectively, these findings suggest that fingerprint-based learning models can reconstruct not only discriminative features, but also emergent, continuous structures that resemble higher-order perceptual organization in the olfactory domain.

## Conclusions

This study demonstrates the effectiveness of various ML models in predicting fragrance categories, with particular emphasis on the superior performance of the structural (ST) model that utilizes 2D Morgan fingerprints derived from 3D MolBlock geometries. Among all tested models, the ST-based approach consistently achieved the highest predictive accuracy across diverse odor labels, owing to its ability to encode detailed substructural information and to learn chemically meaningful patterns directly from molecular topology. Unlike FG models, which rely on predefined SMARTS patterns and human-curated rules, the ST model leverages data-driven fingerprints to represent a broader and more nuanced range of substructures, including chemically relevant motifs that may not be explicitly defined. This expressive capacity enhances its generalizability and makes it particularly suitable for the structure-based discovery of novel fragrance molecules.

A critical factor underlying this performance is the quality of the training data. This study prioritized official descriptors from the IFRA, which are curated to reflect olfactory properties. When IFRA data were available for a compound, no other descriptors were used. For compounds not listed in the IFRA database, fragrance experts meticulously mapped alternative descriptors to the IFRA taxonomy. This rigorous standardization reduced semantic ambiguity and enabled more reliable model training across all feature types, including FG, MD, and ST.

In particular, the latent scent space derived from MDS projection of UMAP-embedded fingerprint vectors reveals a continuous and chemically meaningful spatial organization of odor descriptors. The spatial layout—highlighting both familiar fragrance clusters and plausible transitions among food-related descriptors—suggests that the model captures perceptual and chemical relationships in a continuous manner. This suggests that the ST model serves not only as a high-performing predictive tool but also as a data-driven and interpretable framework for advancing our understanding of the mechanisms underlying olfactory perception. As one of the few studies conducted within an industrial fragrance R&D setting, this work connects academic modeling approaches with practical needs and lays a foundation for structure-guided fragrance design and data-driven olfactory research in perfumery, cosmetics, and flavor industries.

## Supplementary information

Supplementary Information

Description of Additional Supplementary Files

Supplementary Data 1

Supplementary Data 2

Supplementary Data 3

Supplementary Data 4

## Data Availability

**Data Repository:** All datasets used in this study are publicly available via the Open Science Framework (OSF): https://osf.io/vfru6/. Supplementary Data 1 reports classification metrics for all odor labels across three feature types (FG, MD, ST) and three classifiers (RF, XGB, LGBM). Labels with insufficient samples are included but marked as “NaN” where evaluation was not feasible. Supplementary Data 2 provides average feature importance scores for functional group and molecular descriptor models, aggregated across the three tree-based classifiers. Supplementary Data 3 summarizes raw value distributions and model-optimized exemplar values for the three key molecular descriptors—MolLogP, MolWt, and TPSA—used in the MD models. For each odor label, it includes the minimum, maximum, and mean values observed in the dataset, along with the descriptor values of the compound with the highest predicted probability from the MD-XGB model. Supplementary Data 4 contains the 1024-dimensional fingerprint importance vectors for each odor label (Sheet 1), the pairwise distance matrix after UMAP embedding (Sheet 2), and the corresponding 2D MDS coordinates used to generate Fig. 4 (Sheet 3).

## Code availability

All codes in this work are publicly available via Open Science Framework (OSF) (https://osf.io/vfru6/).
