# Supplementary material for: A comparative study of machine learning models on molecular fingerprints for odor decoding
Source: Commun Chem. 2025 Sep 25;8:278. doi: 10.1038/s42004-025-01651-7 (PMC12462479; doi:10.1038/s42004-025-01651-7)
Supplement: Supplementary file 1 — Supplementary Information [file 42004_2025_1651_MOESM1_ESM.pdf]

communications chemistry

Supplementary information for
A comparative study of machine learning models on molecular fingerprints for odor decoding

Jinyoung Suh[1*], Yeonju Hong[1], Chunho Park[1]
[1]R&I Center, COSMAX Inc., 255 Pangyo-ro, Bundang-gu, Seongnam-si, Gyeonggi-do 13486, Republic of Korea.
* Corresponding author: jyseo@cosmax.com

Supplementary Table 1. SMARTS [1] Patterns for Functional Group Detection.

**Functional Group**	**SMARTS Pattern**	**Functional Group**	**SMARTS Pattern**
Hydroxyl	[OH]	Ketone	C(=O)C
Acetate	C(=O)[O]C	Aldehyde	[CX3H1](=O)[#6]
Carbonyl	[CX3]=[OX1]	Alcohol	[CX4][OH]
Carboxyl	C(=O)O	Thiol	[SH]
Amine	[NX3;H2,H1;!$(NC=O)]	Disulfide	[S][S]
Amide	C(=O)N	Sulfide	[SX2]
Nitrile	C#N	Sulfoxide	[SX3](=O)
Ether	[OD2]([#6])[#6]	Sulfonamide	S(=O)(=O)N
Phenol	c1ccc(O)cc1	Sulfonate	S(=O)(=O)[O-]
Ester	C(=O)OC	Halide	[F,Cl,Br,I]

Supplementary Table 2. Five-fold Cross-Validation Performance of Feature Set and Classifier Combinations (≥30 Samples)

Feature set	Classifier	Accuracy	AUROC	AUPRC	Specificity	Precision	Recall
FG	LGBM	0.703	0.744	0.092	0.701	0.056	0.681
	RF	0.739	0.731	0.086	0.738	0.060	0.631
	XGB	0.977	0.742	0.092	0.998	0.057	0.020
MD	LGBM	0.951	0.794	0.184	0.96	0.167	0.302
	RF	0.968	0.734	0.170	0.983	0.210	0.197
	XGB	0.976	0.795	0.182	0.994	0.279	0.118
ST	LGBM	0.962	0.801	0.224	0.972	0.225	0.296
	RF	0.974	0.784	0.215	0.990	0.291	0.171
	XGB	0.978	0.816	0.226	0.995	0.358	0.143

Supplementary References

1. James CA, Weininger D: Daylight Theory Manual. Daylight Chemical Information Systems. Inc. of Aliso Viejo, CA, USA, http://www.daylight.com.
