# Supplementary material for: A comparative study of machine learning models on molecular fingerprints for odor decoding
Source: Commun Chem. 2025 Sep 25;8:278. doi: 10.1038/s42004-025-01651-7 (PMC12462479; doi:10.1038/s42004-025-01651-7)
Supplement: Supplementary file 2 — Description of Additional Supplementary Files [file 42004_2025_1651_MOESM2_ESM.pdf]

Description of Additional Supplementary Files

File name: Supplementary Data 1

Description: Classification metrics for all odor labels across feature types and classifiers. Supports main Figure 1.

File name: Supplementary Data 2

Description: Average feature importance scores for FG and MD models. Supports main Figure 2.

File name: Supplementary Data 3

Description: Raw distributions and model-optimal values for three MD descriptors (MolLogP, MolWt, TPSA). Supports main Figure 3.

File name: Supplementary Data 4

Description: Contains the 1024-dimensional fingerprint importance vectors (one per odor label), UMAP-embedded coordinates, and the corresponding 2D MDS projection values. Supports main Figure 4.
